# UAV Based Indoor Localization and Objection Detection

**DOI:** 10.3389/fnbot.2022.914353

**Published:** 2022-07-08

**Authors:** Yimin Zhou, Zhixiong Yu, Zhuang Ma

**Affiliations:** ^1^Shenzhen Institute of Advanced Technology, Chinese Academy of Sciences, Shenzhen, China; ^2^University of Chinese Academy of Sciences, Beijing, China

**Keywords:** visual SLAM, self-positioning, real-time localization, convolutional neural network, target detection, UAV

## Abstract

This article targets fast indoor positioning and 3D target detection for unmanned aerial vehicle (UAV) real-time task implementation. With the combined direct method and feature method, a method is proposed for fast and accurate position estimation of the UAV. The camera pose is estimated by the visual odometer *via* the photometric error between the frames. Then the ORB features can be extended from the keyframes for the map consistency improvement by Bundle Adjustment with local and global optimization. A depth filter is also applied to assist the convergence of the map points with depth information updates from multiple frames. Moreover, the convolutional neural network is used to detect the specific target in an unknown space, while YOLOv3 is applied to obtain the semantic information of the target in the images. Thus, the spatial map points of the feature in the keyframes can be associated with the target detection box, while the statistical outlier filter can be simultaneously applied to eliminate the noise points. Experiments with public dataset, and field experiments on the established UAV platform in indoor environments have been carried out for visual based fast localization and object detection in real-time for the efficacy verification of the proposed method.

## 1. Introduction

Unmanned aerial vehicles (UAVs) have been developed rapidly in recent years, with diversified applications from military to civil fields, i.e., police patrolling, urban management, agriculture spraying, geology exploration, electric power patrolling, rescue and disaster relief, video shooting, and other industries due to their small size, low cost, high maneuverability, and fast speed (Chebrolu et al., 2018). As a common positioning system, GPS would lose function and fail to provide accurate position information in indoor or GPS-denied environments, hence visual SLAM (Simultaneous Localization And Mapping) technology can be adopted in such positioning scenes owing to its abundant positioning information and wide applicability (Kasyanov et al., 2017). It should be aware that the collected image information from UAV are 2-dimensional (2D) without stereoscopic information, so the “what” of the target can be obtained while the “where” of the target in the space is unknown. In order to complete such post-disaster rescuing tasks successfully under complex, unknown environments, two fundamental problems should be solved simultaneously, UAV self-localization and target detection (Cavaliere et al., 2017).

Visual localization is generally realized *via* visual SLAM technologies, where the visual odometry (VO) (Jiang et al., 2018) can be used to estimate the pose variation of the camera *via* captured consecutive frames, usually divided into two categories, i.e., indirect/feature-based methods and direct methods (Su and Cai, 2018). The most representative of the feature methods is the ORB-SLAM (Mur-Artal et al., 2015), with the aid of ORB (Oriented FAST and Rotated Brief) feature possessing rotation invariance and scale invariance *via* pyramid construction so as to assist SLAM algorithm to have endogenous consistency in the feature extraction and tracking, keyframe selection, 3D reconstruction, and closed-loop detection. Bundle Adjustment (BA) is performed to minimize the feature reprojection error in a local set of keyframes. Furthermore, the localization precision can be improved in ORB-SLAM3 (Campos et al., 2021), where the pose and map points are optimized *via* cumulative errors avoidance at the back-end. However, ORB-SLAM would fail to track in a less texture environment due to insufficient feature points extraction. Unlike feature-based methods, the direct method establishes the relationship between frames through the gray information of pixels to construct the camera motion estimation with a faster processing speed. The direct sparse odometry (DSO) system is further proposed to optimize the photometric parameters for robustness improvement (Wang et al., 2017). However, the cumulative error is inevitable due to a lack of global back-end optimization, resulting in overall poor system accuracy.

Combining the advantages of the direct methods and feature-based methods, a fusion scheme SVO (semi-direct visual odometry) is proposed (Forster et al., 2014), where the minimized photometric error is used to optimize the pose estimate. Moreover, only feature points are extracted from the keyframes for tracking without descriptors calculation for considerable speed enhancement. However, the initialization of the pose could fail in the head-up view if all the points are not on the same plane and SVO lacks global optimization. Then the 2nd version of SVO2.0 has been proposed by adding multi cameras to improve the tracking of edges and corners and IMU (internal measurement unit) pre-integration (Forster et al., 2016), which can further increase the processing speed. Although vision-fused IMU can enhance the robustness of the SLAM system, it would also bring higher algorithm complexity and degrade the real-time performance.

Object detection is dependent on the image understanding, mainly including two parts, i.e., type decision and size and position estimate of the object in the image. Since AlexNet (Beeharry and Bassoo, 2020) won the championship in ILSVRC2012, a deep convolutional neural network (CNN) has widely been applied with autonomously learning features. RCNN (region CNN) (Girshick et al., 2014) is a pioneering work of applying deep CNN to target detection. FastRCNN (Girshick, 2015) and FasterRCNN (Ren et al., 2016) are further proposed to effectively avoid the image scaling problem. On the other hand, YOLO (You Only Look Once), has been proposed in Redmon et al. (2016), which can directly regress multiple positions of the image to acquire the target box and category, thus simplifying the detection process. Combined with YOLO and FasterRCNN, a new algorithm SSD (single shot multibox detector) (Liu et al., 2016) is proposed to obtain the frame coordinates *via* different convolutional layers. To date, YOLOv5 is released in Oct 2020, possessing higher object identification accuracy (Kuznetsova et al., 2020).

Different from 2D target detection, 3D target detection should mark the spatial position of the target. In Chen et al. (2017), a set of 3D object proposals with stereo images for 3D object detection are generated by minimizing an energy function that encodes several depth-informed features, i.e., prior object size, object placement on the ground plane as an extension of FastRCNN in 3D field. Although the proposed method can outperform in object detection and orientation estimation tasks for all three KITTI (database issued by Karlsruhe Institute of Technology and Toyota Technological Institute) object classes, it has a low processing speed per image up to 4s with worse real-time performance. A stereo 3D object detection method is proposed with Instance-DepthAware module and disparity adaptation and matching cost reweighting in Peng et al. (2020), where solely RGB (Red-Green-Blue) images are used as the training data to predict the depth of the 3D bounding boxes centering in the images. Although image-based methods have achieved great success in object detection, the performance of 3D object detection falls behind the LiDAR-based (Light Detection And Ranging-based) approaches due to the inaccurate depth information. While the image-based depth maps can be converted to pseudo-LiDAR representation *via* transformation from the dense pixel depths of stereo imagery and back-projecting pixels into a 3D point cloud (Chen et al., 2020), the main challenge is the heavy computation load of the LiDAR-based detection.

The combination of the visual SLAM and object detection can increase the environment perception capability. For example, VSO (visual semantic odometry) (Liu H. et al., 2019) can optimize the reprojection error between images through semantic information. A semantics SLAM system (Lee et al., 2019) is proposed with the combination of sensor status and semantic landmarks which can transform the semantic map into a probability problem to optimize the reprojection error. The system robustness can also be improved *via* the intensity-SLAM (Wang et al., 2021) and loop detection (Liu Y. et al., 2019) optimization. Moreover, SOF-SLAM (Cui and Ma, 2019) can identify the dynamic features through semantic optical flow and remove these features, achieving stable tracking in dynamic scenes.

Visual SLAM can assist in target detection as well (Vincent et al., 2020). A semantic fusion method (Li et al., 2018) is proposed to combine CNN with a dense vision SLAM scheme for semantic segmentation, where the category probability distributions of the images are fused into a SLAM map to construct a 3D dense semantic map. Further, the semantic information acquirement efficiency can be greatly increased with the adoption of the SSD or YOLO framework (Bavle et al., 2020). For instance, Quadric SLAM (Nicholson et al., 2018) is proposed to identify the position, size and direction of the object simultaneously. However, this kind of method usually requires semantic segmentation of the target based on dense pixel or point cloud information.

**Figure 1:**
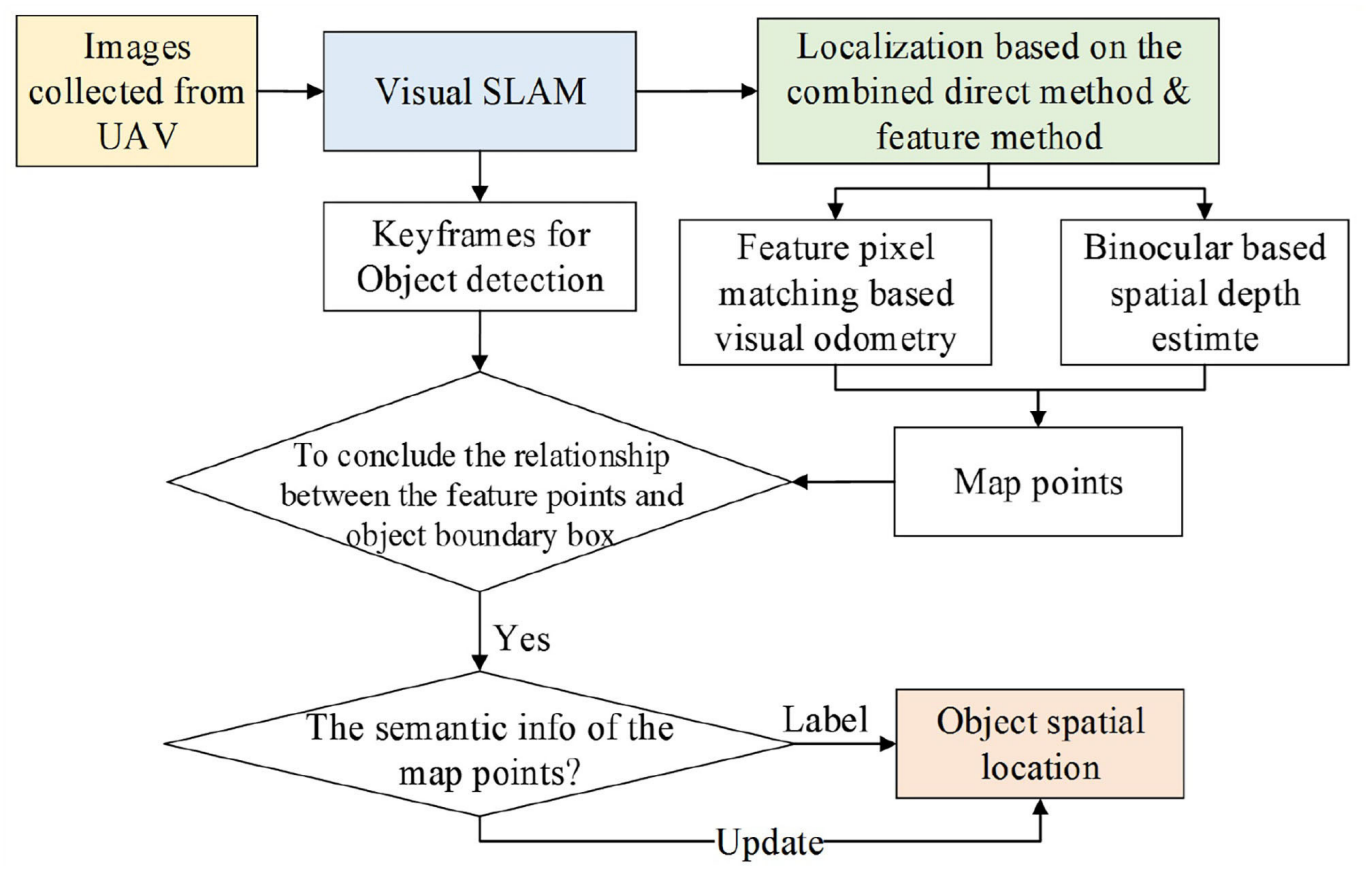
The procedure of the proposed method.

The current target detection algorithms can only estimate the target position in the images, while the spatial position of the target still remains unknown. To tackle the mentioned problems during UAV indoor localization, this article proposes an object localization framework for object spatial location estimation (refer to Figure 1). To be specific, the positioning method is designed to achieve rapid and accurate location estimation of the UAV. Then the target spatial position estimation based on CNN is applied to detect the searched target in an unknown space. The contributions of the article are summarized as follows.

1. A method is proposed for UAV fast self-positioning. Applying the direct method with a visual odometer delicately, the optical error is used for inter-frame matching to estimate the camera posture. The ORB feature extracted from the keyframes can improve the map consistency while a binocular depth filter is introduced to increase the positioning accuracy.
2. A method is proposed for target spatial position estimation based on YOLOv3. The spatial position of the target can be constructed by correlating the relationship among the feature points for reliable depth information providence and the target detection frame in the keyframes. Furthermore, the statistical outlier filter is used to eliminate the noise to acquire more accurate target position.
3. Unmanned aerial vehicle platform has been setup for indoor rapid positioning and object detection. A series of experiments on the public dataset and in the actual scene have been performed to verify the effectiveness of the proposed method with real-time target spatial localization performance.

The remainder of the article is organized as follows. Section II explains the method of indoor localization in detail. Section III explains the objection detection and spatial position estimation. In Section IV, the experiment platform of the indoor UAV fast localization and objection detection is setup for the proposed method verification with public data and field scenarios. Section V concludes the article and future directions are provided.

## 2. Method of Indoor Localization

**Figure 2:**
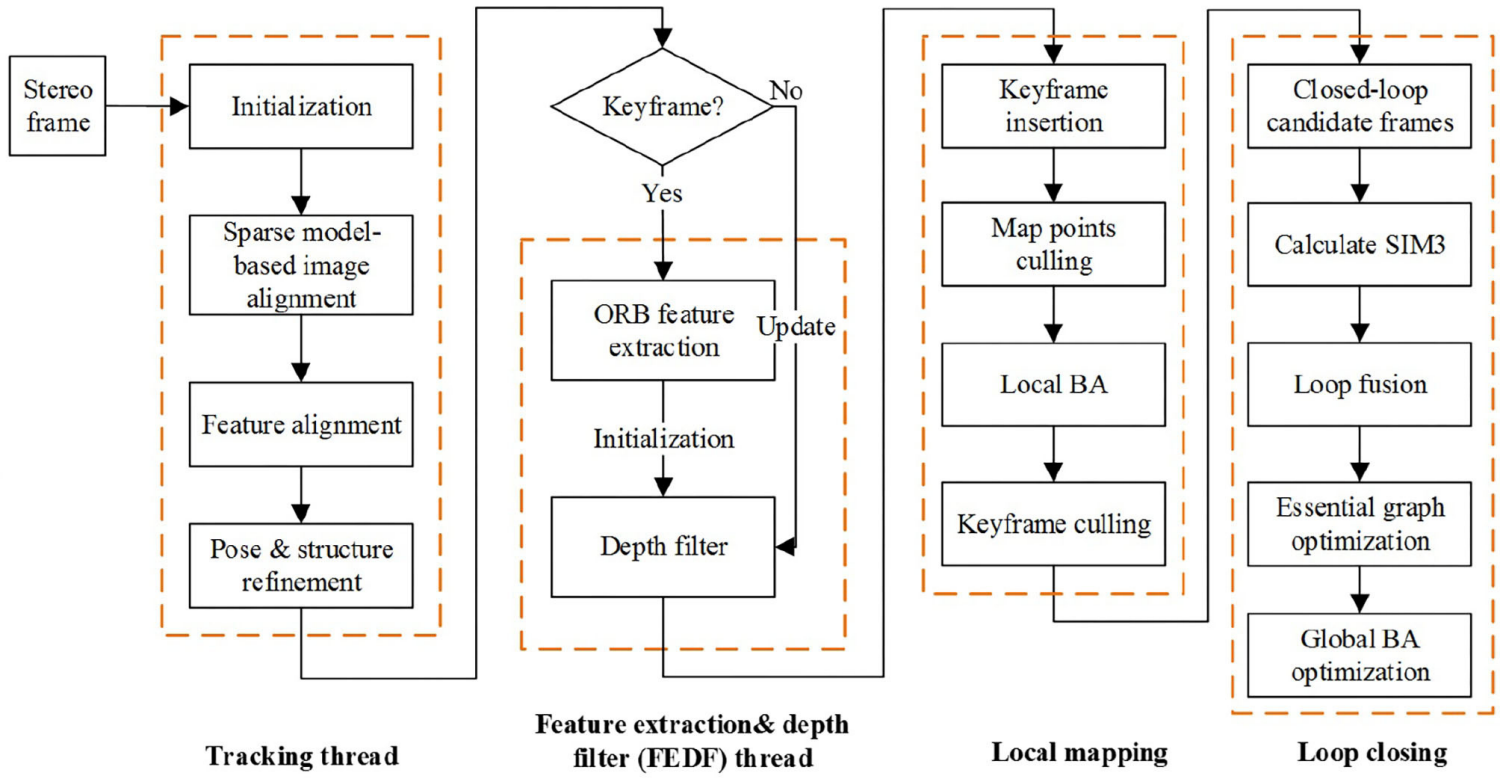
The framework of the proposed localization algorithm.

Combined with the direct method and feature-based method, a localization algorithm is proposed in this Section. Here, ORB-SLAM2 rather than ORB-SLAM3 algorithm is adopted with only an embedded stereo camera for the localization and the whole procedure is depicted in Figure 2. The localization algorithm includes four threads, i.e., tracking thread, feature extraction and depth filter (FEDF) thread, local mapping thread, and loop-closing thread.

The VO based on the direct method for localization in the tracking thread is the first proposed algorithm while the second algorithm for spatial point depth estimation is designed on the basis of stereo depth filter embedded in the FEDF thread. The other two local mapping and loop closing threads are kept the same as in ORB-SLAM2.

### 2.1. Direct Method Based Visual Odometer

Since the VO in ORB-SLAM2 has to extract feature points and calculate descriptors per frame, SVO is adopted for direct frame matching *via* feature points. The procedure of the direct method based VO contains three steps: pose estimate, feature points alignment and pose optimization, where the coordinate origin of the coordinate axes is the left lens optical center of the stereo camera.

### 2.2. Step I. Direct Method Based Pose Estimation

**Figure 3:**
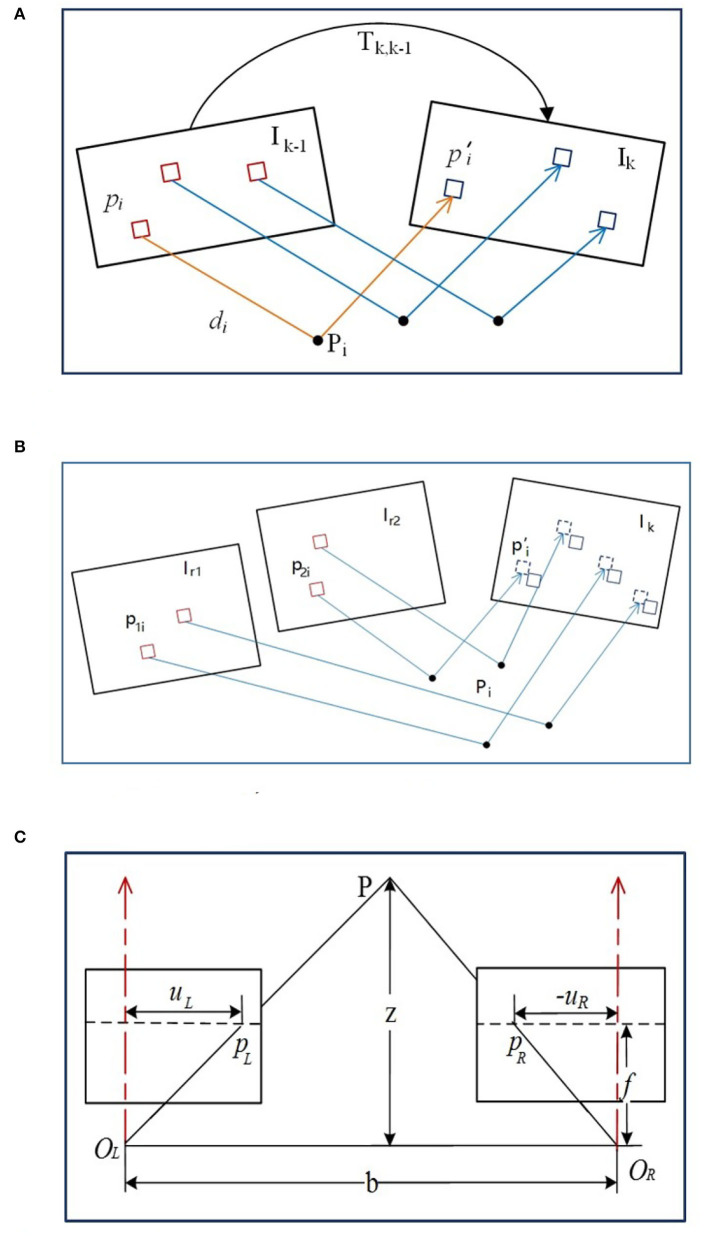
The illustration of image measurement. **(A)** The direct method based pose estimation. **(B)** The alignment of feature pixels. **(C)** Triangulation measurement with stereo camera.

First, the feature points of the binocular image are extracted till the number exceeds the threshold, then the image is designated as the keyframe. Next, the parallax is obtained by binocular image feature matching and the map point depth can be obtained with triangulation so as to acquire the initial pose and map points position. As depicted in Figure 3A, {*I*_*k*_, *I*_*k*−1_} are the image intensities at the *k*^*th*^ moment and the previous (*k* − 1)^*th*^ moment, while *T*_*k,k*−1_ describes the pose variant from the (*k* − 1)^*th*^ moment to the *k*^*th*^ moment. π is used to describe the projection process from 3D space to the image space, while π^−1^ is the back projection process.

The initial pose of the *T*_*k,k*−1_ can be obtained *via* the uniform velocity model or the identity matrix, while the feature point coordinate and the related spatial depth of the (*k* − 1)^*th*^ frame can be estimated from the previous multiple frames, denoted as (*p*_*i*_, *d*_*i*_). Then the specific feature point can be projected at the spatial point *p*_*i*_ with the coordinate *P*_*i,k*−1_ in the (*k* − 1)^*th*^ frame reference. Through the *T*_*k,k*−1_ transformation, it can be converted with the *k*^*th*^ frame reference, denoted as *P*_*i,k*_. Afterwards, it can be projected on the *k*^*th*^ image *via* camera model with the coordinate $p_{i}^{\prime }$.

The brightness of the same point in the two consecutive frames is assumed unchanged due to the transient time interval (Jiang et al., 2018). Thus, the residual function can be formed based on the gray value difference of the image patches adjacent the (*k* − 1)^*th*^ feature point and the reprojected point of the *k*^*th*^ frame,


(1) $$\begin{array}{l} \delta I\;(T_{\text{k},\text{k}-1},p_{i})=I_{k}\;(\pi \cdot \left(T_{\text{k},\text{k}-1}\cdot \pi^{-1}\;(p_{i},d_{\text{i}})\right))-I_{k-1}\;(p_{i}) \end{array}$$

where δ*I* (*T*_k,k−1_, *p*_*i*_) is the intensity variation due to the pose movement from the (*k* − 1)^*th*^ moment to the *k*^*th*^ moment. Then the pose variation in *T*_*k,k*−1_ transformation can be optimized *via* the maximum likelihood estimation by minimizing the photometric residual,


(2) $$\begin{array}{l} T_{\text{k},\text{k}-1}=\arg\underset{T_{\text{k},\text{k}-1}}{\min}\frac{1}{2}\underset{i\in R}{\sum }{\left\|\delta I\;(T_{\text{k},\text{k}-1},p_{i})\right\|}^{2} \end{array}$$

where *R* is the visible image points set in the *k*^*th*^ frame back-projected from the points with the known depth *d*_*i*_ in the image at the (*k* − 1)^*th*^ moment. Gauss-Newton (G-N) or Levenberg-Marquadt (L-M) iterative methods (Balabanova et al., 2020) can be used to solve Equation (2) for the update of *T*_*k,k*−1_ estimation.

### 2.3. Step II. Alignment of the Feature Pixels

With the obtained pose *T*_*k,k*−1_ between two consecutive frames *via* direct method, the feature points from the previous frame (*k* − 1) can be reprojected on the current frame *k* but with coordinate inconsistency due to the noise. Since more accurate pixel information exists in the common view keyframe adjacent to the current frame *k*, the position of the feature pixels in the *k* frame can further be optimized through the established map points in the nearest common view keyframes. The pose relationship among the *k* frame and the common view keyframes *I*_*r*1_ and *I*_*r*2_ can be acquired from *T*_*k,k*−1_, so the feature points *p*_1*i*_ and *p*_2*i*_ in the keyframes can be reprojected on the current frame *k*, illustrated in Figure 3B. Assuming luminosity invariance, the residual function can be reconstructed from the image gray values difference to optimize the coordinates of feature points *via* minimized luminosity error,


(3) $$\begin{array}{l} p_{i}^{\prime }=\arg\underset{p_{i}^{\prime }}{\min}\frac{1}{2}{\left\|I_{k}\;(p_{i}^{\prime })-A_{i}\cdot I_{r}\;(p_{i})\right\|}^{2} \end{array}$$

where *I*_*r*_(·) is the previously observed frame, *A*_*i*_ is the rotating and stretching affine operation. If the common view keyframes are far away from the current frame, the feature patch in the keyframe should be first transformed *via A*_*i*_ operation for further comparison. Again, Equation (3) can be solved *via* G-N or L-M methods for more accurate coordinates estimate $p_{i}^{\prime }$ obtainment.

### 2.4. Step III. Pose Optimization

With a more precise match between the features of the keyframe and the previous frame, the feature points can be reprojected on the current *I*_*k*_ frame. Then the position residual function is formed by the pixel coordinate difference from the reprojection point and the related $p_{i}^{\prime }$, written as,


(4) $$\begin{array}{l} \left\|\delta p_{i}\right\|=\left\|p_{i}^{\prime }-\pi \cdot \left(T_{\text{k},\text{w}}P_{i}\right)\right\| \end{array}$$

and the pose of the current frame is optimized as,


(5) $$\begin{array}{l} T_{\text{k},\text{w}}=\arg\underset{T_{\text{k},\text{w}}}{\min}\frac{1}{2}\underset{i}{\sum }{\left\|p_{i}-\pi \cdot \left(T_{\text{k},\text{w}}P_{i}\right)\right\|}^{2} \end{array}$$

while the position *P*_*i*_ on the map can also be optimized *via* the same maximum likelihood function simultaneously with G-N or L-M methods for solution.

### 2.5. Binocular Based Spatial Point Depth Estimation

Building an accurate and reliable map is necessary for the camera pose calculation from the spatial map points *via* feature matching and triangulation. Since the map point depth from the triangulation is highly affected by the parallax from the two frames, a depth filter is introduced for depth optimization with the adoption of the calibrated stereo camera for the initial seed point depth determination.

#### 2.5.1. Triangulation Ranging Model

The stereo camera model should be rectified first, usually with epipolar line adjustment (Bradski and Kaehler, 2008) to make the optical axes parallel with *O*_*L*_ and *O*_*R*_ centers. As shown in Figure 3C, *b* is the baseline of the stereo camera, *f* is the focal length, *p*_*L*_ and *p*_*R*_ are the positions of the spatial point *P* in the left and right cameras, and {|*u*_*L*_|, |*u*_*R*_|} are the distances from each axis of {*p*_*L*_, *p*_*R*_}. Based on the similar triangles, it has,


(6) $$\begin{array}{l} \frac{z-f}{z}=\frac{b-u_{L}+u_{R}}{b},\;x=u_{L}-u_{R}\Rightarrow \;z=\frac{fb}{x} \end{array}$$

where *x* is the parallax, i.e., the difference of binocular abscissa, and *z* is the spatial depth. The depth usually follows the normal distribution (Ammann and Mayo, 2018), i.e., the depth *z*_*p*_ of the spatial point *P* follows *N* (μ, σ^2^) distribution. With the depth *z*_*k*_ (followed by $N\;(\mu_{o},\sigma_{o}^{2})$ normal distribution) calculated *via* epipolar line matching from the new *k*^*th*^ frame, new depth data are fused with the existing ones, still followed by normal distribution $N\;(\mu_{f},\sigma_{f}^{2})$,


(7) $$\begin{array}{l} \mu_{f}=\frac{\sigma_{o}^{2}\mu +\sigma^{2}\mu_{o}}{\sigma^{2}+\sigma_{o}^{2}},\;\sigma_{f}^{2}=\frac{\sigma_{o}^{2}\sigma^{2}}{\sigma^{2}+\sigma_{o}^{2}} \end{array}$$

**Figure 4:**
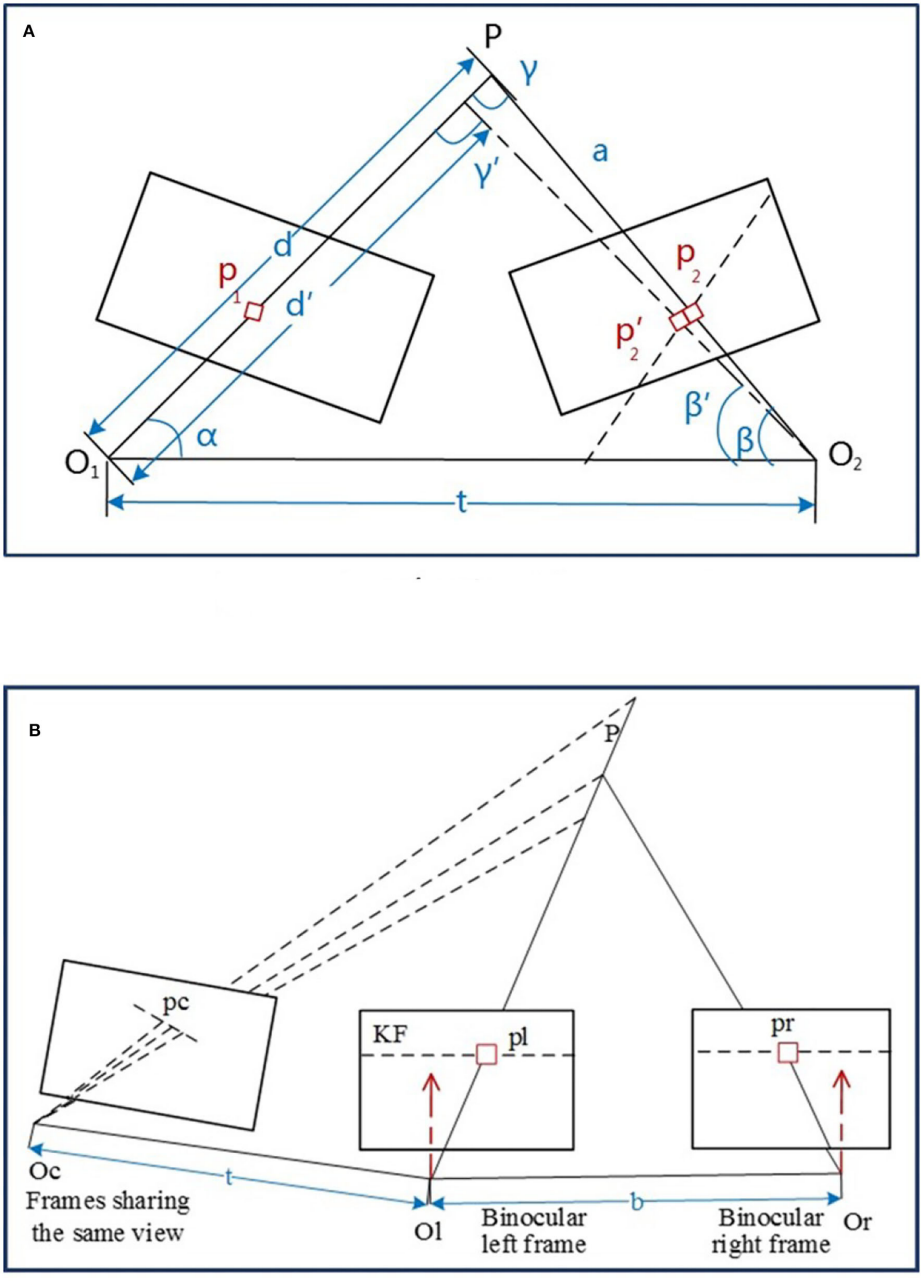
The illustration of depth estimation. **(A)** Depth estimate with uncertainty. **(B)** Spatial point depth estimate.

The depth can be assumed as converged if σ_*f*_ is less than the threshold, whereas the uncertainty of the depth should also be considered. Given a spatial point *P*, it is projected on *p*_1_ and *p*_2_ of any two frames with the optical centers *O*_1_ and *O*_2_, respectively, as depicted in Figure 4A.

The pixel error of the spatial point *P* is considered as the distance variation and the related angles changed from {||*d*||, {*p*_2_, β, γ}} to {||*d*′||, $\left\{p_{2}^{\prime },\beta^{\prime },\gamma^{\prime }\right\}\}$. Based on the geometric relationship, it has,


(8) $$\begin{array}{c} \vec{\alpha }=\vec{d}-\vec{t}\\ \beta =arccos<\vec{d},\;\vec{t}>\\ \alpha =arccos<\vec{\alpha },\;\vec{-t}> \end{array}$$

It can be rearranged as,


(9) $$\begin{array}{l} \left\|d^{\prime }\right\|=\left\|t\right\|\frac{\sin\beta^{\prime }}{\sin\gamma^{\prime }} \end{array}$$

where β is the angler between *O*_2_*P* and *O*_1_*O*_2_, and γ is the angler between *O*_1_*P* and *O*_1_*O*_2_. Thus, the depth uncertainty caused by one pixel bias is,


(10) $$\begin{array}{l} \sigma_{o}=\left\|d\right\|-\left\|d^{\prime }\right\| \end{array}$$

Since the inverse depth also obeys the normal distribution (Younes et al., 2019), the σ_*o*_ variance can be transformed as


(11) $$\begin{array}{l} \sigma_{o}=\left\|\frac{1}{d}\right\|-\left\|\frac{1}{d^{\prime }}\right\| \end{array}$$

Finally, with the pose calculation of the new frame, the depth filter is used to merge the depth and the depth uncertainty into the previous ones until the uncertainty is less than the threshold so as to generate the map points accordingly.

#### 2.5.2. Depth Estimation

The depth filter is initialized with the calculated depth from feature matching of the extracted ORB features from the keyframe. Given a point *P*, the left and right image frames can be triangled *via* Equation (6) to obtain their initial depth values (seen in Figure 4B). The point *P* is assumed as the joint view with a non-keyframe and keyframe, while the new estimated depth can be calculated by matching through the feature block mean gradient and epipolar line searching so that the depth filter can be updated.

Furthermore, the newly added spatial points are fused with the existing map points. The minimized reprojection error function is constructed to optimize the pose of the keyframe and the coordinates of the spatial points. Finally, the redundant keyframes are filtered out and sent to the loop-closing thread.

Here, the loop-closing thread is the same as ORB-SLAM2 for similarity comparison of the bag of words of the new keyframes and existing ones. If the similarity level exceeds a dynamic threshold, a closed-loop occurs and the pose of the new keyframe and the closed-loop keyframe are adjusted so that the poses of all the keyframes are optimized. With the relocation and loopback ability of ORB-SLAM2, the lost camera's pose tracking can be recovered *via* the position comparison of the feature points among the previous keyframes and the current frame. At the same time, a more accurate trajectory and map can be obtained *via* closed-loop fusion with the data from different tracking periods.

## 3. Object Detection and Position Estimation

### 3.1. Algorithm Framework

As general object detection algorithms, the target type and position in the images can be estimated *via* salient feature detection. However, especially in complex scenarios, only the 2D target position in the image is insufficient without the 3D target spatial position. Although the constructed semantic map combined with the objection detection and visual SLAM can be used to estimate the object category and position in a 3D environment, it is mostly achieved based on dense or semi-dense point clouds for multiple targets estimation, which has a higher requirement on the collection and calculation. As for the mission of UAV in indoor environments, there is no need to rebuild the 3D mapping environment or estimate the detailed posture of the target but only require to label the location of the target in the searching paths. Hence, object detection and spatial location estimate to the specific targets are proposed based on the sparse space points.

The YOLOv3 object detection framework based on CNN is adopted in this article, which is integrated into the visual SLAM system. The keyframes are sent to the YOLOv3 object detection framework to detect the specific target, i.e., people, and the spatial map points stored in the keyframes are used to construct the relationship between the detected target and the spatial point position, so as to estimate the spatial position of a specific target. The details of the YOLOv3 are omitted here, readers who are interested in the algorithm can refer to Zhang et al. (2020).

### 3.2. The Estimate of the Target Spatial Position

**Figure 5:**
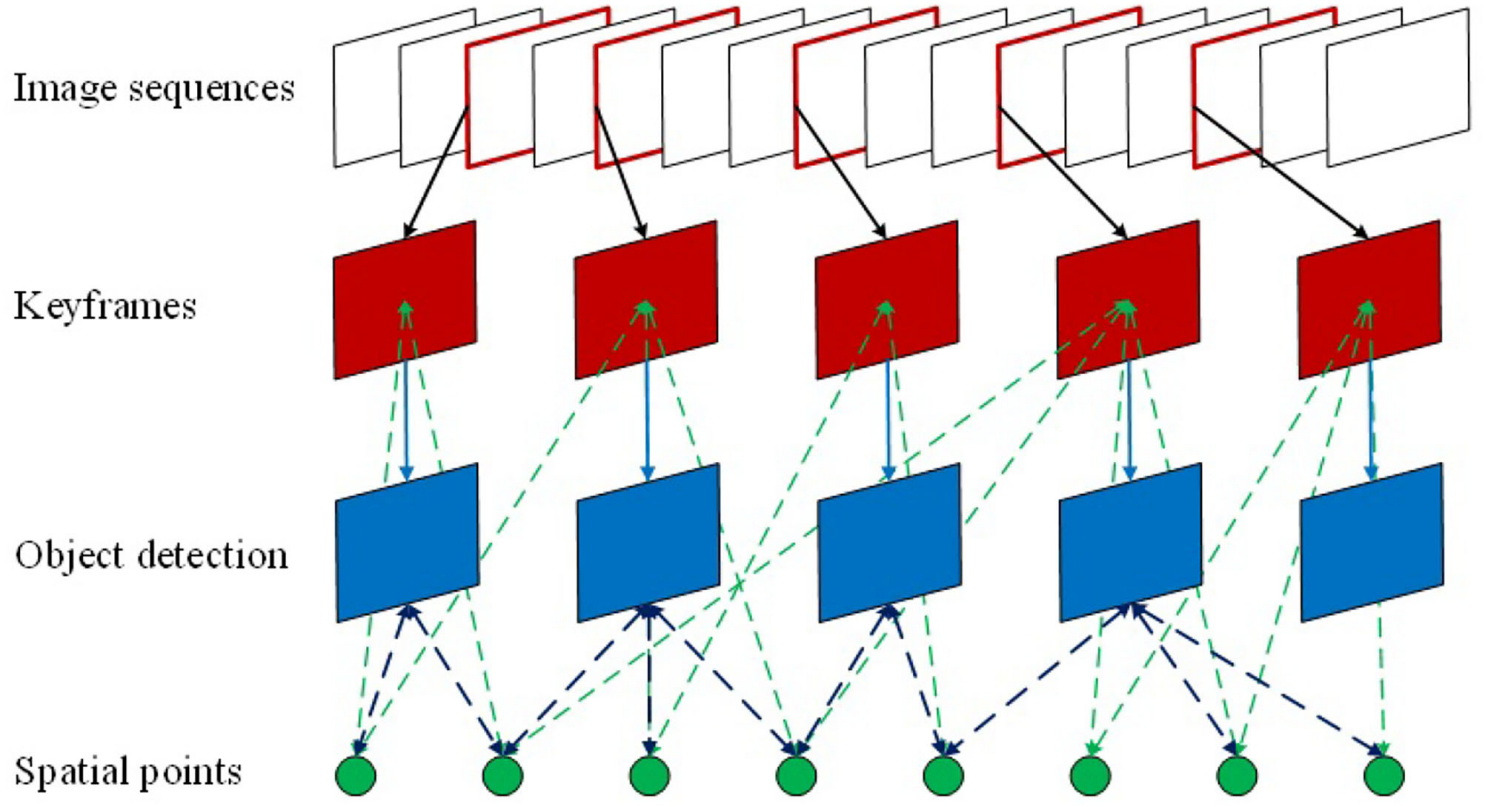
The relationship between the object and spatial map points.

As seen in Figure 5, since the map points information of SLAM is kept in the keyframes, the feature points in the keyframes can thus be connected with the objects *via* the object detection so as to estimate the spatial location of the target.

**Figure 6:**
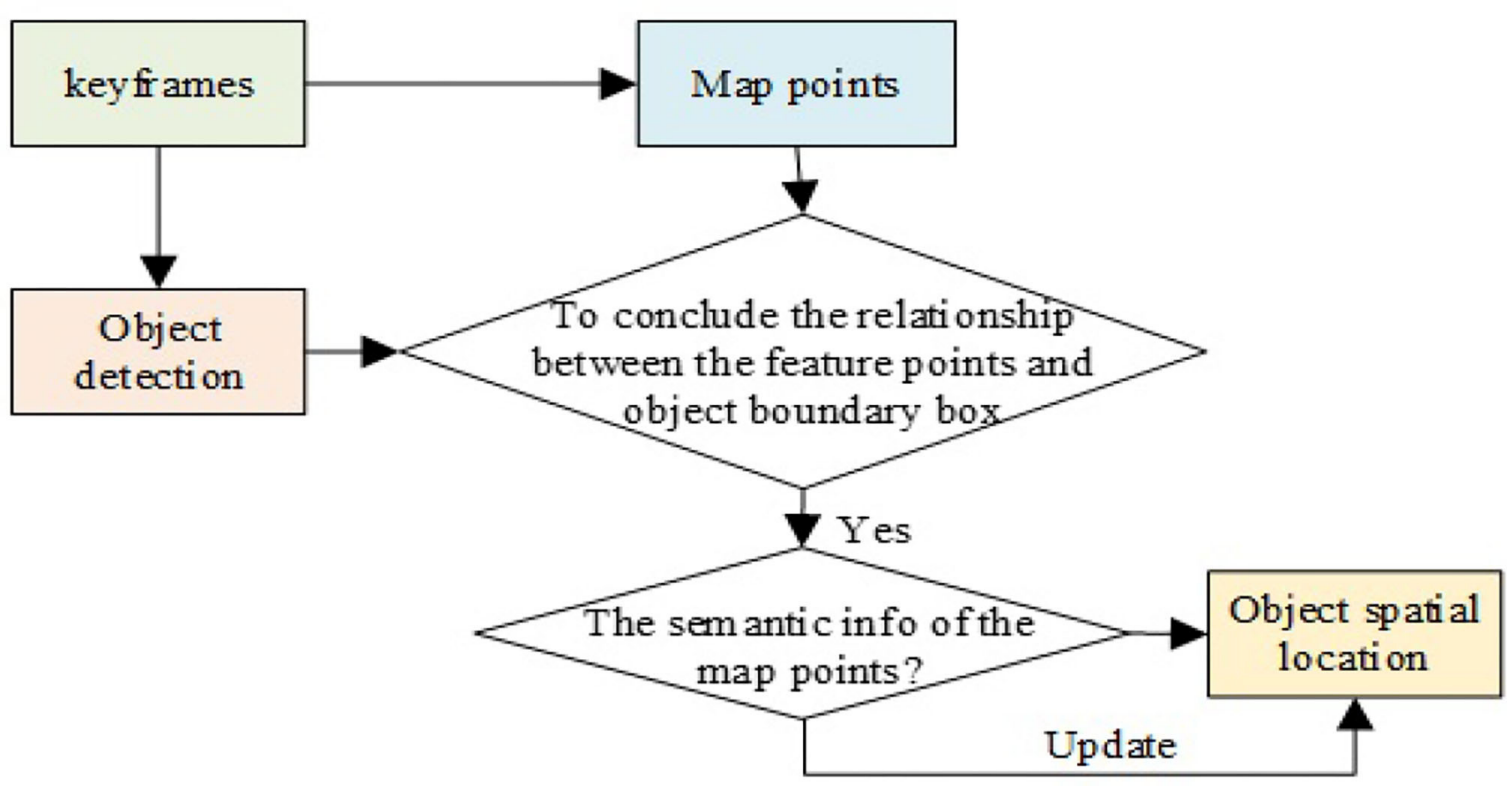
The procedure of the spatial target position estimation.

First, the keyframe images are sent to the YOLOv3 network for object detection, which is used to determine the relationship between the feature points in the keyframes and the object detection frame. If the feature point is inside the detection boxes, the map points related to the feature points are searched to identify whether the map points have already been marked with the target semantic information. If not, it will be marked as a new pending target and if it is marked as the same pending target in 3 consecutive frames, the point is thus marked as a new spatial target point. The world coordinate is applied here as the reference system to calculate the maximum distance of the spatial target point distribution in the *x*, *y*, and *z* directions, where these distances are used as the widths of the target bounding box to mark the spatial target position in the object detection box. Subsequently, the ratio of the map points is calculated carrying semantic information to all map points in the bounding box. When the ratio exceeds the threshold, the semantic information of the map points related to other feature points in the box is updated as the new semantic information, together with the spatial target position and the detection box. The procedure of the spatial object location estimation is illustrated in Figure 6.

**Figure 7:**
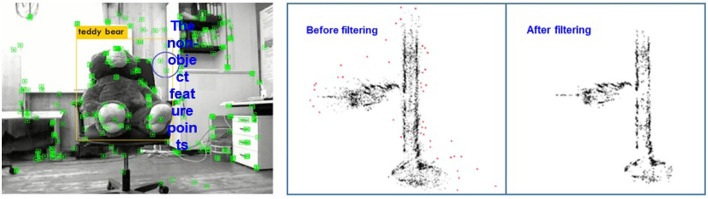
(Left): The position illustration of the target box and the feature points; (Right): The result of the statistical outlier filter.

The object detection box in the detection algorithm is usually a rectangle, which cannot accurately represent the boundary between the target area and the background area as background points are often included inside the target box resulting negative effect on the accuracy of the target spatial position estimation. For instance, the target rectangle box in the left graph of Figure 7 not only contains the target “teddy bear” but also the feature points of the background wall.

Generally speaking, the distances between the background points and the target points are large, and the number of background feature points in the detection box is less than that of the target. Therefore, the depth information of the feature points in the object detection rectangle can be filtered by the spatial point distribution. Here, SOF (Statistical Outlier Filter) is adopted to remove the outliers (Bokovoy and Yakovlev, 2018).

As for any spatial point, the mean distance to its nearest *k* points can be calculated. Assuming the mean distance of each point follows the normal distribution with the expected average value μ and variance σ^2^, the threshold of the mean distance is written as,


(12) $$\begin{array}{l} d_{max}=\mu +\alpha \times \sigma \end{array}$$

where α is the scale factor of neighboring points. Therefore, the points whose distances between the neighboring points are larger than the defined threshold is removed (refer to the right graph of Figure 7) so as to estimate the position of the target in space more accurately.

## 4. Experiments for Indoor UAV Fast Localization and Object Detection

### 4.1. Experiments of Localization on Public Data

The public UAV dataset EuRoC (ASL, 2012) is adopted here containing indoor sequences collected from an AscTecFirefly micro UAV where the resolution of the stereo camera is 752 × 480 and 20*fps* (frame per second) processing speed. EuRoC data includes 11 sequences, while the sequences {{MH01, MH02, MH03}, V201} are randomly selected from the large industrial workshop and general office room such as two typical scenes. Besides, another real trajectory collected by the Leica MS50 lasers canner is used for performance comparison. The processing environment of the experiments is Ubuntu 16.04 system, GPU is Nvidia GeForce GTX 1080, and the processor is Inteli7-8750 with 16GB RAM. Then the proposed method is evaluated from the processing time per frame and pose localization accuracy in two criteria indexes.

#### 4.1.1. Processing Time Evaluation

**Figure 8:**
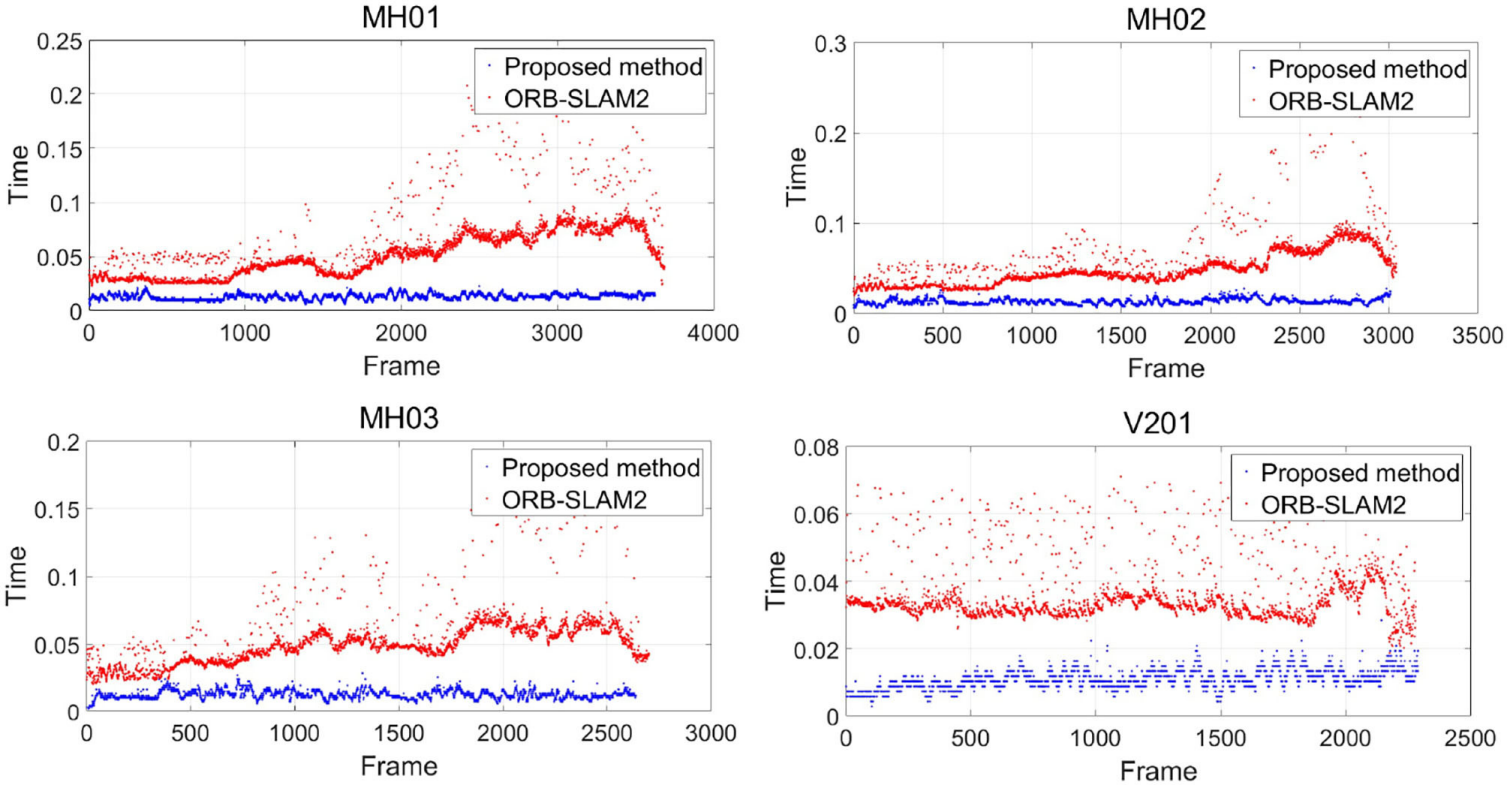
Image processing time distribution per frame.

Figure 8 shows the distribution of processing time spent for each frame, while the horizontal coordinate is the frame number, and the vertical coordinate is the processing time in seconds. The red points and blue points delegate the processing rate of ORB-SLAM2 and the proposed method, respectively. It is shown in Table 1 that the frame processing speed of our method is much faster than that of the ORB-SLAM2 with four random selected tracks, which is primarily due to the direct matching with the photometric error during tracking with no feature extraction and descriptor calculation.

**Table 1:** The processing time per frame.

**Data**	**Number of the frames**	**Proposed method(s)**	**ORB-SLAM2(s)**
MH01	3,682	0.0128	0.0539
MH02	3,040	0.0129	0.0502
MH03	2,700	0.0127	0.0511
V201	2,280	0.0111	0.0357

#### 4.1.2. Position Accuracy Evaluation

**Figure 9:**
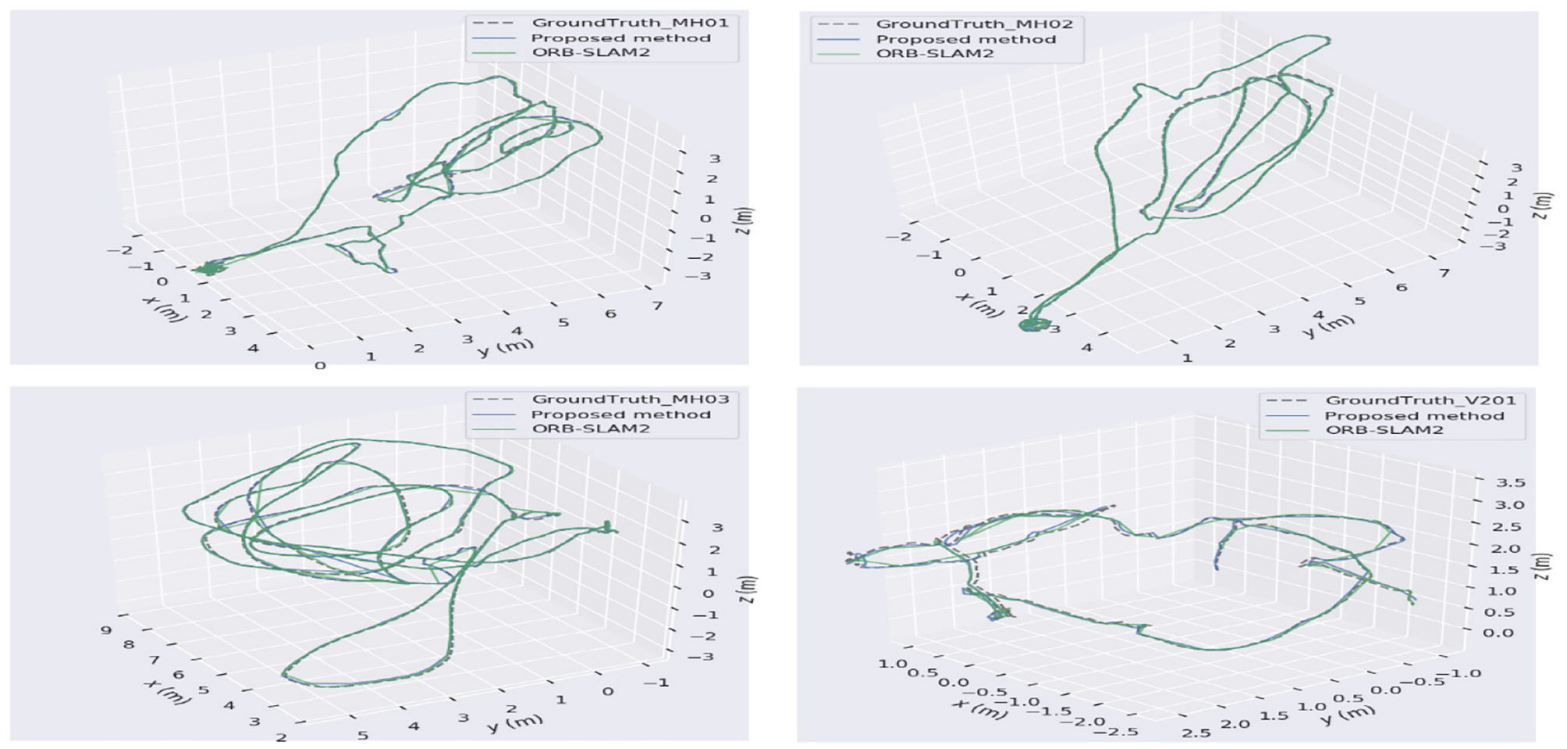
Keyframe trajectory comparison on EuRoc datasets.

Due to the difficulty in measuring actual 3D map points, the track error of the camera motion is generally used for VO or visual SLAM algorithm performance evaluation. Figure 9 illustrates the trajectories comparison among the four datasets and the actual one from the proposed algorithm and ORB-SLAM2, denoted as the blue curve, green curve, and dashed line, respectively. To be specific, the Root Mean Square Error (RMSE) is used for location evaluation between the estimate and the actual one,


(13) $$\begin{array}{l} RMSE=\sqrt{\frac{1}{n}\overset{n}{\underset{i}{\sum }}{\left({\hat{X}}_{i}-X_{i}\right)}^{2}} \end{array}$$

where *X*_*i*_ is the real data and ${\hat{X}}_{i}$ is the estimate, *n* is the group number of the data. “Max” and “Mean” represent the maximum and mean discrepancy between the estimates and the real data. Then the numerical comparison results are listed in Table 2. As demonstrated in Table 2, the pose estimate accuracy is nearly the same as the proposed method and the ORB-SLAM2, which is due to the introduction of depth filter to optimize the depth of the spatial points during map point location estimation, allowing the position of the map points with higher accuracy. On the other hand, it can improve the pose estimation accuracy while the back-end optimization based on keyframes can enhance the localization precision as well. Therefore, the whole location precision is higher or the same compared to ORB-SLAM2.

**Table 2:** Keyframe trajectory accuracy comparison(m).

	**Proposed method**			**ORB-SLAM2**		
	**Max**	**Mean**	**RMSE**	**Max**	**Mean**	**RMSE**
MH01	0.0902	0.0274	0.0347	0.0810	0.0307	0.0363
MH02	0.0900	0.0276	0.0331	0.1012	0.0312	0.0375
MH03	0.1112	0.0338	0.0378	0.1071	0.0391	0.0421
V201	0.1182	0.0588	0.0613	0.0964	0.0392	0.0458

### 4.2. Experiments of Target Detection on Public Data

#### 4.2.1. Image Object Detection

**Figure 10:**
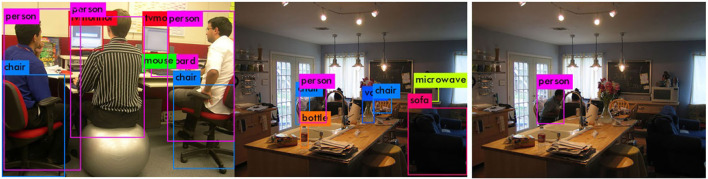
The result of category detection. **(A)** Multi-category detection. **(B)** Single-category detection.

The original YOLOv3 network is used to detect various object types, but only people are required to be detected under this circumstance. In order to mitigate the impact of other objects, the original network is retrained only for the human category. The images selected from the public data set VOC2012 (PASCAL, 2012) are used for comparison between the original YOLOv3 and the modified network, where the involved parameters setting in YOLOv3 are listed in Table 3. Figure 10 displays the detection results where the modified network can well detect the expected objects.

**Table 3:** The parameters involved in YOLOv3.

**Paramter**	**Value**	**Parameter**	**Value**
Batch	64	Exposure	1.5
Subdivisions	16	hue	0.1
Width	416	Learning-rate	0.001
Height	416	Burn-in	1,000
Channels	3	Max-batches	50,200
Momentum	0.9	Policy	steps
Decay	0.0005	Steps	40,000, 45,000
Angle	0	Scales	0.1, 0.1
Saturation	1.5		

#### 4.2.2. Object Spatial Position Estimation

**Figure 11:**
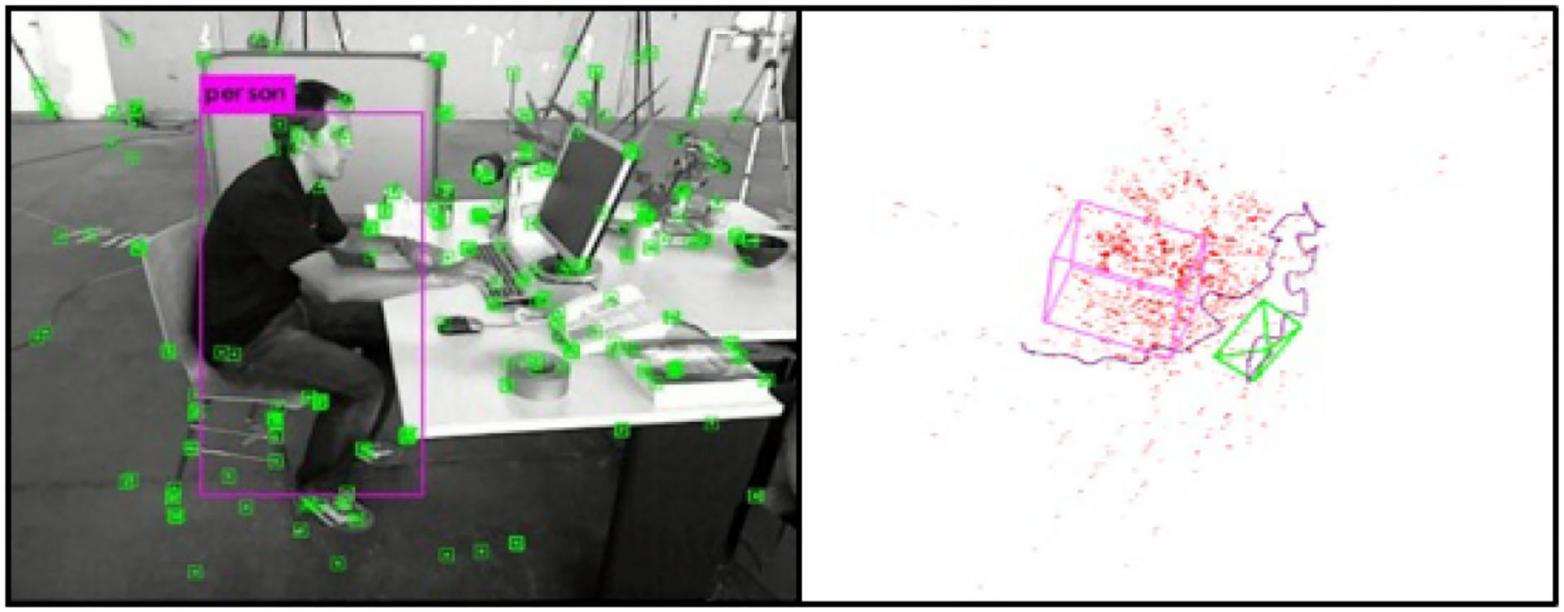
Estimation results of the object spatial position.

After the object is detected in the image, it is necessary to associate the object semantic information with the map points to estimate the spatial position of the object and mark it on the map. In this article, the public data set TUM (TUM, 2020) is used to test the object spatial position estimation. The Tum data set is provided by the Technical University of Munich, which includes RGBD, monocular, 3D reconstruction, and other various experimental scenes. In order to meet the requirement of indoor scenes containing the object (people) to be detected, *freiburg*2−*desk*−*with*−*person* data set is selected for verification (refer to Figure 11) with a resolution of 640 × 480 and average *fps* 28.6fps.

It is known that the relationship between the object detection frame and the image feature point can be constructed by detecting the specific object (people) in the keyframes by YOLOv3, so as to establish the relationship between the object and the spatial map point and estimate the object spatial position on the map. As shown in the left graph of Figure 11, the human object can be detected *via* the YOLOv3 model with the extracted feature points in the keyframe images. The result of the spatial object position estimation is demonstrated in the right graph of Figure 11, including the spatial point cloud, camera running trajectory, and the cubic mark of the detected human. Since the direction of the spatial object frame in this article is based on the world coordinate system when the camera is initialized, the spatial object frame is not completely consistent with the direction of the image detection frame, but the relative spatial position of the object remains unchanged.

### 4.3. Field Experiments

**Figure 12:**
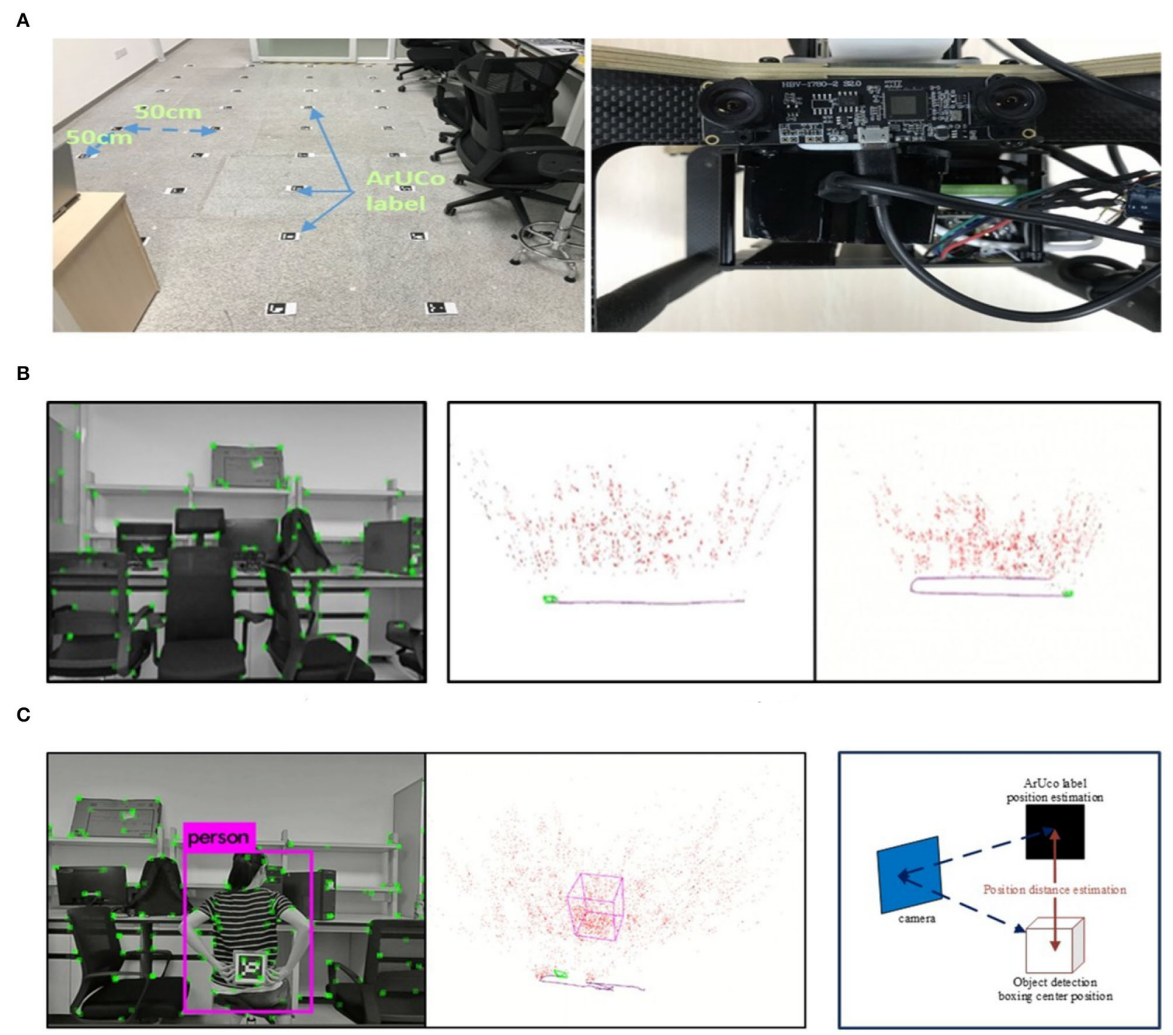
The illustration of the indoor test procedure and results. **(A)** The experimental scene and binocular camera. **(B)**The tracking screenshot in the actual scene. **(C)** (Left): The object detection in the actual scene; (Right): Comparison of object position estimation.

The UAV platform in the real world has been well set up for fast localization and objection detection, which is configured with Pixhawk as the controller, together with main processor 32bit STM32F427 Cortex M4, and other embedded sensors. The experimental environment is an indoor laboratory scene, with a length of about 7m and a width of about 6m, mainly including tables, chairs, and several office supplies. The manually set labels are equally distributed on the ground with a 0.5m interval as the positioning reference, shown in Figure 12A. In the experiments, the quadrotor UAV equipped with a binocular camera is used as the experimental platform, where the resolution rate is 2, 560 × 720, the frame processing rate is 30 fps, and the length of the binocular baseline is 60 mm. In order to facilitate the comparison tests, the collected binocular video is saved and converted into a data set, which is analyzed offline.

#### 4.3.1. Indoor Positioning Test

The indoor positioning test is mainly evaluated with the processing time per frame and the positioning accuracy through the collected dataset. The dataset consists of two tracks, and the actual running screenshot is shown in the left graph of Figure 12B, while the middle track is the linear motion and the right track is the linear rotary motion.

With these two tracks, the processing rate is compared between the proposed algorithm and ORB-SLAM2. Table 4A lists the average processing time per frame under the two track segments. It can be seen that the proposed algorithm has a faster processing speed than that of the ORB-SLAM2 in actual scenes.

**Table 4:** The performance comparison of the tracking algorithms.

**(A) AVERAGE PROCESSING TIME (S) PER IMAGE FRAME**			
	**Proposed algorithm**	**ORB-SLAM2**	
Track 1	**0.0158**	0.0457	
Track 2	**0.0186**	0.0560	
**(B) TRACKING ACCURACY COMPARISON(M)**			
	**Proposed algorithm**		
	**Max**	**Mean**	**RMSE**
Track 1	0.0484	0.0309	0.0326
Track 2	0.0730	0.0433	0.0467

*The bold values indicate the indexes of the proposed algorithm*.

In order to acquire the positioning reference of the UAV flight position, ArUco tags with different IDs are equally distributed on the ground at 50 cm distance in the experimental environment (refer to the left graph in Figure 12C). The actual position of each ArUco tag in the world coordinate can be obtained by the ID of each ArUco tag, and the captured ArUco image is used to estimate the position of the ArUco tag, so as to estimate the camera position in the world coordinate accordingly.

The image of ArUco tag is captured by a vertically downward high-definition camera installed on the UAV, which has a resolution of 1, 920 × 1, 080 and frame processing rate 60 fps, fixed relative to the binocular camera. It is difficult to obtain the complete trajectory pose due to certain unrecognized phenomena in the moving process. Then the posture of ArUco on the path is used as the baseline for evaluation, while the results listed in Table 4B demonstrate that the proposed algorithm can realize real-time localization with high precision.

#### 4.3.2. Object Detection Test

As for the object detection test, a dataset containing the detection object (people) is used for the experiments. The people to be detected are in a still sitting state. Through the UAV moving in the scene, the spatial position of the people can be estimated and marked with a box on the map (refer to Figure 12C of the actual running screenshot). Since the object detection is based on the keyframes, only the object detection results and the object position estimation from the keyframes are evaluated. In this experiment, the number of keyframes including the object is 112, and the number of keyframes with confirmed object detection is 88, while the recall rate is 78.6%.

As for the estimation of the object spatial position, the object's handheld ArUco tag is used as the comparison benchmark. With the confirmed keyframes inclusion, the relative position of the tag in the keyframes can be estimated *via* the identification of the handheld ArUco tag, shown as the tag held by the people in the left graph of Figure 12C. Whereas in the same keyframe, the relative position between the center of the spatial object frame and the current keyframe can also be obtained, compared with the relative position estimated by the ArUco tag, as shown in the right graph of Figure 12C. Then three keyframes are randomly selected where the distance difference between the two estimated positions is used for comparison, while the results of the estimated distance difference (m) of the three keyframes are {0.96, 1.04, 0.88}, respectively.

Through the evaluation of the detection recall rate and the object spatial position estimation, it can be concluded that the object can be detected effectively with more accurate spatial position estimation in actual scenes, which is suitable for real-time task implementation.

## 5. Conclusion

In this article, a real-time rapid positioning and object detection method based on UAV has been explored with the combination of visual SLAM and CNN techniques. Considering the advantages of feature-based methods with VO, a fast positioning algorithm is proposed where the camera pose can be tracked *via* the front-end VO with only ORB features extracted from the keyframes for the purpose of map consistency improvement *via* bundle adjustment. The feature-based method is also applied at the back-end with the depth filter to assist the depth convergence of the map points so as to optimize the framework for positioning accuracy improvement. Furthermore, a spatial target position estimation algorithm has been proposed with the CNN in an unknown space, while the YOLOv3 network is also applied for the target semantic info obtainment in the images so as to construct the relationship between the spatial points and the target. Moreover, the spatial noise can be removed from a statistical outlier filter so as to acquire a clearer target boundary. A series of experiments with public datasets and field tests have been performed to verify the accuracy and portability of the still object localization method with only embedded UAV hardware processor for surveillance or rescuing such task execution, especially in GPS-denied environments.

Future work will continue to study the UAV posture estimation with appropriate semantic segmentation and IMU modules to improve the robustness and accuracy of the UAV fast localization. More efficient signal filtering algorithms could be developed to remove the spatial noise in the key features. Besides, the target attitude estimate should be investigated to increase the localization accuracy since the actual orientation of the target has not been considered. The proposed localization method will be testified in more harsh field experiments.

## Data Availability Statement

The raw data supporting the conclusions of this article will be made available by the authors, without undue reservation.

## Author Contributions

ZY and YZ contributed to conception and design of the study. ZY organized the database and performed the main experiments and wrote the first draft of the manuscript. ZY, YZ, and ZM corrected the sections of the manuscript. All authors contributed to manuscript revision, read, and approved the submitted version.

## Funding

This work was supported under National Natural Science Foundation of China (NSFC) (61973296, U1913201), STS project of Chinese Academy of Sciences (KFJ-STS-QYZX-107), Shenzhen Science and Technology Innovation Commission Project Grant (JCYJ20170818153635759), and Shenzhen Fundamental Research Program (JCYJ20200109114839874).

## Conflict of Interest

The authors declare that the research was conducted in the absence of any commercial or financial relationships that could be construed as a potential conflict of interest.

## Publisher's Note

All claims expressed in this article are solely those of the authors and do not necessarily represent those of their affiliated organizations, or those of the publisher, the editors and the reviewers. Any product that may be evaluated in this article, or claim that may be made by its manufacturer, is not guaranteed or endorsed by the publisher.
